# Semi-Classical Discretization and Long-Time Evolution of Variable Spin Systems

**DOI:** 10.3390/e23060684

**Published:** 2021-05-28

**Authors:** Giovani E. Morales-Hernández, Juan C. Castellanos, José L. Romero, Andrei B. Klimov

**Affiliations:** Departamento de Física, Universidad de Guadalajara, Guadalajara 44420, Jalisco, Mexico; giovani.morales8917@alumnos.udg.mx (G.E.M.-H.); juan.castellanos@alumnos.udg.mx (J.C.C.); jose.romero@cucei.udg.mx (J.L.R.)

**Keywords:** phase-space, semiclassical evolution, variable spin systems, 03.65.Ta, 03.65.Sq, 03.65.Fd

## Abstract

We apply the semi-classical limit of the generalized SO(3) map for representation of variable-spin systems in a four-dimensional symplectic manifold and approximate their evolution terms of effective classical dynamics on $T^{*}S_{2}$. Using the asymptotic form of the star-product, we manage to “quantize” one of the classical dynamic variables and introduce a discretized version of the Truncated Wigner Approximation (TWA). Two emblematic examples of quantum dynamics (rotor in an external field and two coupled spins) are analyzed, and the results of exact, continuous, and discretized versions of TWA are compared.

## 1. Introduction

Phase-space methods provide a very convenient framework for the analysis of large quantum systems (QS) [1,2,3,4]. In cases when the states of QS are elements of a Hilbert space H which carries a unitary irreducible representation of a Lie group *G*, trace-like maps from operators $\hat{f}$ acting in H into their (Weyl) symbols $W_{f}\left(\Omega \right)$ can be established. The symbols $W_{f}\left(\Omega \right)$ are functions on the corresponding classical phase-space M, Ω∈M being the phase-space coordinates. The most suitable in applications is frequently the Wigner (self-dual) map, allowing the average value of an observable to be computed as a convolution of its symbol with the symbol of the density matrix $W_{\rho }\left(\Omega \right)$ (the Wigner function). The properties of the Wigner function are extremely useful for studying the quantum-classical correspondence. In particular, the quantum evolution is described by a partial differential equation (the Moyal equation [5]) for the Wigner function with a well-defined classical limit. In the non-harmonic case, the Moyal equation contains high-order derivatives, which turns the finding of its exact solution into quite a difficult task.

The advantage of the phase-space approach to quantum dynamics consists of the possibility of expanding the Moyal equation in powers of small parameters in the semi-classical limit. Such semi-classical parameters are related both to the symmetry of the Hamiltonian and of the map.

In general, different mappings can be performed for composite quantum systems. The type of map fixes the structure of the phase-space manifold, and thus the allowed set of shape-preserving transformations. In addition, the phase-space symmetry determines the physical semiclassical parameter ε≪1.

The standard phase-space approach [6,7,8,9,10] is not applicable for the construction of covariant (under group transformations) invertible maps if the density matrix cannot be decomposed into a direct sum of components each acting in an irreducible representation of the dynamical group *G*. Physically, this happens when the Hamiltonian of the system induces transitions between irreducible subspaces i.e., the total angular momentum is changed in the course of evolution. This happens, for instance, in quantum systems with non-fixed (variable) spin, such as large interacting spins, a rigid rotor in an external field, coupled and externally pumped boson modes with and without decay, etc.

A suitable SU(2) covariant map, establishing a one-to-one relation between operators and set of functions (discretely labelled symbols) in a three-dimensional space, can be found for variable spin systems [11]. In addition, there exists a continuous limit of such symbols for large values of the mean spin [12]. This allows the quantum operators to be put in correspondence with smooth functions on the four-dimensional cotangent bundle $T^{*}S_{2}$, equipped with a symplectic structure. In particular, the semi-classical dynamics of the Wigner function of variable spin systems can be described in terms of effective “classical” trajectories $\Omega^{cl}\left(t\right)$ in the phase-space $T^{*}S_{2}$. As a rough approach, the evolution of average values can then be estimated within the framework of the so-called Truncated Wigner Approximation (TWA), which consists of propagating points of the initial distribution along the classical trajectories. Such an approximation has been successfully applied for studying a short-time evolution of QS with different dynamic symmetry groups [13,14,15,16,17,18,19,20,21,22,23,24,25,26,27]. Expectably, the TWA fails to describe the non-harmonic evolution beyond the Ehrenfest (or semiclassical) time $\tau_{sem}$, [28,29,30,31,32], where intrinsic quantum correlations effects start to emerge. The semi-classical time heavily depends both on the Hamiltonian and on the initial state (in particular on the stability of the classical motion). For instance, for spin *S* systems, the semiclassical time usually scales as the inverse power of the spin length, $gt≲S^{-\alpha }$, α>0, where *g* is a constant characterizing the non-harmonic dynamics. The semiclassical time for variable spin systems behaves in a similar way, where the effective spin size is proportional to the average total angular momentum.

Multiple attempts to improve the TWA [19,33,34] suggest that more sophisticated methods [35,36,37,38,39,40,41,42,43] should be applied in order to describe the quantum evolution in terms of continuously distributed classical phase-space trajectories. Alternatively, different types of discrete phase-space sampling were proposed [44,45,46,47,48,49,50] in order to emulate evolution of average values using the main idea of the TWA. In general, phase-space discretization is a tricky question, which has been addressed from different perspectives mainly focusing on the flat and torus phase-space manifolds [51,52].

It is worth noting that the phase-space analysis of some variable spin systems, such as, for example, two large interacting spins, can be formally performed using the Schwinger representation. Then, faithful mapping onto a flat $C^{2}⊗C^{2}$ phase-space is carried out by applying the standard Heisenberg-Weyl H(1)×H(1) map [53,54,55,56]. However, the natural SU(2) symmetry is largely lost in such an approach. This is reflected, in the fact that (a) the corresponding distributions are not covariant under rotations; and (b) the inverse excitation numbers in each of the boson modes play the role of *dynamical* semi-classical parameters. The explicit time-dependence of such semi-classical parameters may restrict the validity of the formal division of the Moyal equation to the classical part, containing only the Poisson brackets and the so-called “quantum corrections”. All of this leads to inefficiency of the standard semiclassical methods [19]. The map of the same systems onto the $S_{2}⊗S_{2}$-spheres in the Stratonovich–Weyl framework [57,58,59] reveals only the local SU(2) symmetry. The semiclassical parameters are the inverse spin lengths (constant in time). Thus, the SU(2) TWA leads, in principle, to better results than its flat counterpart in $C^{2}⊗C^{2}$. However, the standard discretization of the $S_{2}$ sphere [60] does not lead to a significant improvement of the TWA, since the location of the initial distribution is not taken into account.

The situation is even more intricate when the number of involved invariant subspaces becomes formally infinite (or physically very large), as, for example, in the case of a highly-excited rigid rotor interacting with external fields. The use of the Schwinger representation leads to substantial complications in both the analytical and numerical calculations and the standard SU(2) map is simply non-applicable. Thus, the analysis of the semi-classical limit becomes very challenging [61,62].

In the present paper, we show that there is a natural discretization of $T^{*}S_{2}$ in the vicinity of the initial distribution, which allows the time-scale of validity of the TWA to be significantly increased, including the so-called revival times. Such a discretization is based on the asymptotic form of the star-product for spin-variable systems [12] and is directly applicable to calculations of the evolution of mean values of physical observables. Using our approach, we will be able to describe the long-time dynamics of molecule in an external field (modelled by a physical rotor) and a coupled two-spin system in the semi-classical limit. We restrict our study to quantum systems with SO(3) symmetry, corresponding to integer spins.

The paper is organized as follows: In Section 2, we recall the basic results on the SO(3) covariant mapping for variable spin systems. In Section 3, we discuss the asymptotic form of the star-product in the semi-classical limit. In Section 4, we develop a discretization scheme on the classical manifold of variable spin systems and apply it to computation of mean values in a “quantized” version of the TWA. Two applications of the proposed method with the corresponding numerical solutions are discussed in Section 5. A summary and conclusions are given in Section 6.

## 2. Variable Spin Wigner Function

Let us consider a QS whose states are elements of a Hilbert space H containing multiple SO(3) irreps, so that the SO(3) group, in general, does not act irreducibly on the density matrix of the system $\hat{\rho }$, i.e.,

$$\hat{\rho }=\sum_{L,L^{\prime }=0,1,..}\sum_{m,m^{\prime }}c_{mm^{\prime }}^{LL^{\prime }}|L,m\rangle \langle L^{\prime },m^{\prime }|.$$

The generalized Wigner-like map [11] from operators acting in H to a discrete set of functions, later called *j*-symbols,

(1) $$\hat{f}\Leftrightarrow \left\{W_{f}^{j}\left(\Theta \right);\;j=0,1,\cdots \right\},$$

where

(2) $$\Theta =\left(\phi ,\theta ,\psi \right)\in S_{3},\;0\leq \phi <2\pi ,\;0\leq \theta <\pi ,\;0\leq \psi <2\pi$$

are the Euler angles, is defined through a trace operation

(3) $$W_{f}^{j\;}\left(\Theta \right)=\mathrm{Tr}\left(\hat{f}{\hat{\omega }}_{j}\left(\Theta \right)\right),$$

where the Hermitian SO(3) covariant mapping kernels have the form

(4) $${\hat{\omega }}_{j}\left(\Theta \right)=\sum_{K=0}^{j}\sum_{Q,Q^{\prime }=-K}^{K}\sqrt{\frac{2K+1}{j+1}}D_{QQ^{\prime }}^{K}\left(\Theta \right){\hat{T}}_{KQ}^{\frac{j+Q^{\prime }}{2}\;\frac{j-Q^{\prime }}{2}},$$

where $D_{QQ^{\prime }}^{K}\left(\Theta \right)$ is the Wigner *D*-function, $D_{QQ^{\prime }}^{K}\left(\Theta \right)=\langle K,Q|e^{-i\phi {\hat{l}}_{z}}e^{-i\theta {\hat{l}}_{y}}e^{-i\psi {\hat{l}}_{z}}|K,Q^{\prime }\rangle$, here ${\hat{l}}_{x,y,z}$ are generators of SO(3) group, $\left[{\hat{l}}_{k},{\hat{l}}_{m}\right]=i\varepsilon_{kmn}{\hat{l}}_{n}$,

(5) $${\hat{T}}_{KQ}^{JJ^{\prime }}=\sum_{M,M^{\prime }}\sqrt{\frac{2K+1}{2J+1}}C_{J^{\prime }M^{\prime },\;KQ}^{JM}|J,M\rangle \langle J^{\prime },M^{\prime }|,$$

are tensor operators [63] and $C_{a\alpha ,\;b\beta }^{c\gamma }$ are the Clebsch–Gordan coefficients. The map (3) is explicitly invertible

(6) $$\begin{array}{ccc} \hat{f} & = & \sum_{j=0,1\cdots }^{\infty }{\hat{f}}_{j}, \end{array}$$

(7) $$\begin{array}{ccc} {\hat{f}}_{j} & = & \frac{j+1}{8\pi^{2}}\int d\Theta W_{f}^{j}\left(\Theta \right){\hat{\omega }}_{j}\left(\Theta \right), \end{array}$$

where dΘ=sinθdϕdθdψ is a volume element of $SO\left(3\right)$, leading to the overlap relation

(8) $$\mathrm{Tr}\left(\hat{f}\hat{g}\right)=\sum_{j=0,1,2,\cdots }^{\infty }\frac{j+1}{8\pi^{2}}\int d\Theta W_{f}^{j}\left(\Theta \right)W_{g}^{j}\left(\Theta \right).$$

It is worth noting that the operators ${\hat{f}}_{j}$ correspond to the expansion of $\hat{f}$ on the tensor operators ${\hat{T}}_{KQ}^{JJ^{\prime }}$ (5) in the sectors with fixed values of $j=J^{\prime }+J$:

(9) $$\begin{array}{ccc} {\hat{f}}_{j} & = & \sum_{K=0}^{j}\sum_{Q,Q^{\prime }=-K}^{K}{\hat{T}}_{K\;Q}^{\frac{j+Q^{\prime }}{2}\;\frac{j-Q^{\prime }}{2}}f_{K\;Q}^{\frac{j+Q^{\prime }}{2}\;\frac{j-Q^{\prime }}{2}}, \end{array}$$

(10) $$\begin{array}{ccc} f_{K\;Q}^{\frac{j+Q^{\prime }}{2}\;\frac{j-Q^{\prime }}{2}} & = & Tr\left(\hat{f}{\hat{T}}_{K\;Q}^{\frac{j+Q^{\prime }}{2}\;\frac{j-Q^{\prime }}{2}†}\right). \end{array}$$

It should be noted that $Q^{\prime }$ in (4), running over even or odd integers depending on the parity of the index *j*, such that the restriction $Q^{\prime }\pm j$ is an even number, is fulfilled. This leads to the following symmetry properties of the kernel:

(11) $$\begin{array}{ccc} {\hat{\omega }}_{j}\left(\phi ,\theta ,\psi \right) & = & {\hat{\omega }}_{j}\left(\phi ,\theta ,\psi +\pi \right),\;\mathrm{even}\;j, \end{array}$$

(12) $$\begin{array}{ccc} {\hat{\omega }}_{j}\left(\phi ,\theta ,\psi \right) & = & -{\hat{\omega }}_{j}\left(\phi ,\theta ,\psi +\pi \right),\;\mathrm{odd}\;j. \end{array}$$

The advantage of the generalized map (1)–(3) consists of the possibility of a “classical” representation of the whole operator acting in H and not only its projections on the SO(3) irreducible subspaces. For instance, for the orientation operators, $\hat{r}$,

(13) $$\begin{array}{ccc} \hat{r} & = & \int d\varphi \;\sin\vartheta d\vartheta \;n\left(\varphi ,\vartheta \right)|\varphi ,\vartheta \rangle \langle \varphi ,\vartheta |,\;\;{\hat{r}}^{2}=\hat{I}, \end{array}$$

(14) $$\begin{array}{ccc} |\varphi ,\vartheta \rangle & = & \sum_{J=0,1,\cdots }\sum_{M=-J}^{J}Y_{JM}^{*}\left(\varphi ,\vartheta \right)|J,M\rangle , \end{array}$$

where φ, ϑ are angles in the configuration space, $n\left(\varphi ,\vartheta \right)=\left(\cos\varphi \sin\vartheta ,\sin\varphi \sin\vartheta ,\cos\vartheta \right)$, one obtains

(15) $$W_{r_{k}}^{j}\left(\Theta \right)=r_{k}\sum_{n\in Z^{+}}\delta_{j,2n+1},$$

where $r\;=\;\left(\sin\phi \sin\psi -\cos\phi \cos\theta \cos\psi ,-\cos\phi \sin\psi -\sin\phi \cos\theta \cos\psi ,\sin\theta \cos\psi \right)$, $r^{2}=1$. The dependence of the symbol on the angle ψ indicates that the corresponding operator mixes SO(3) invariant subspaces. Vice-versa, symbols of the operators that preserve each SO(3) irreducible subspace are independent of ψ, as, for instance, the angular momentum operators, $\hat{l}=\left({\hat{l}}_{x},{\hat{l}}_{y},{\hat{l}}_{z}\right)$,

(16) $$W_{l_{k}}^{j}\left(\Theta \right)=\sqrt{\frac{j}{2}\left(\frac{j}{2}+1\right)}\;n_{k}\left(\phi ,\theta \right)\sum_{n\in Z^{+}}\delta_{j,2n},$$

where $n\left(\phi ,\theta \right)=\left(\cos\phi \sin\theta ,\sin\phi \sin\theta ,\cos\theta \right)$ is a unitary vector in the parameter space (2), and, as a consequence,

(17) $$W_{l^{2}}^{j}\;\left(\Theta \right)=\frac{j}{2}\left(\frac{j}{2}+1\right)\sum_{n\in Z^{+}}\delta_{j,2n},$$

where ${\hat{l}}^{2}$ is the square angular momentum operator.

The standard Stratonovich–Weyl kernel ${\hat{w}}_{L}\left(\phi ,\theta \right)$ [57,58,59], used for mapping operators acting in a single SO(3) subspace of dimension 2L+1=j+1 (*L* is an integer),

(18) $$\hat{f}\Leftrightarrow W_{f}\left(\phi ,\theta \right)=Tr\left(\hat{f}{\hat{w}}_{L=j/2}\left(\phi ,\theta \right)\right),$$

is recovered from the generalized kernel ${\hat{\omega }}_{j}\left(\Theta \right)$ (4) by integrating over the angle ψ (for even values of *j*):

(19) $${\hat{w}}_{L=j/2}\left(\phi ,\theta \right)=\int_{0}^{2\pi }\frac{d\psi }{2\pi }\;{\hat{\omega }}_{j}\left(\Theta \right)=\sqrt{\frac{4\pi }{2L+1}}\sum_{K=0}^{2L}\sum_{Q=-K}^{K}\;Y_{KQ}^{*}\left(\phi ,\theta \right)\;{\hat{T}}_{KQ}^{L}\;$$

where $Y_{KQ}\left(\phi ,\theta \right)$ are spherical harmonics and ${\hat{T}}_{KQ}^{L}$ are the standard (diagonal) tensor operators [64,65].

It is important to stress that the kernel (4) is not reduced to the direct product of the standard SO(3) kernels (19). Therefore, the map (1), possessing the underlying global SO(3) symmetry, allows us to faithfully represent operators in the form of *c*-functions that:

(a) act in two independent SO(3) irreps, as, for example, a direct product of angular momentum operators ${\hat{l}}_{k}^{\left(1\right)}⊗{\hat{l}}_{m}^{\left(2\right)}$. It should be observed that an alternative mapping can also be achieved with the kernel ${\hat{w}}_{L_{1}}\left(\phi ,\theta \right)⊗{\hat{w}}_{L_{2}}\left(\phi ,\theta \right)$. However, in the latter case, the underlying symmetry group is SO(3)×SO(3). The advantage of one of the map over another is not obvious. It will be shown below that the map (1) admits a natural discretization in the semiclassical limit that significantly improves the range of applicability of the Truncated Wigner Approximation;

(b) mixes all SO(3) irreps, as, for example, the orientation operator (13). This type of operators cannot be mapped into their classical counterparts in the framework of the standard Stratonovich–Weyl approach (18).

## 3. Wigner Function Dynamics in the Semi-Classical Limit

The crucial feature of the map (1)–(3) is the possibility of introducing a star-product operator [5,66], acting on *j*-symbols [11]:

(20) $$W_{fg}^{j}\left(\Theta \right)=\sum_{j_{1},j_{2}=0,1,\cdots }L^{j,j_{1}j_{2}}\left(W_{f}^{j_{1}}\left(\Theta \right)W_{g}^{j_{2}}\left(\Theta \right)\right),$$

which is reduced to the standard (local) form [67,68] when the operators $\hat{f}$ and $\hat{g}$ are operators from the se(3) enveloping algebra. The exact form of the star-product operator in general is non-local on the index *j* and has an involved form, but it is significantly simplified in the limit j≫1 [12,27],

(21) $$\begin{array}{ccc} L^{j,j_{1}j_{2}} & \approx & V\int_{0}^{2\pi }\frac{d\varphi d\varphi^{\prime }}{{\left(2\pi \right)}^{2}}e^{i\left(j_{2}-j+J^{0}⊗I\right)\varphi }e^{i\left(j_{1}-j-I⊗J^{0}\right)\varphi^{\prime }}, \end{array}$$

(22) $$\begin{array}{ccc} V & = & e^{-\varepsilon J^{0}⊗J^{0}-\frac{\varepsilon }{2}\left(J^{+}⊗J^{-}-J^{-}⊗J^{+}\right)}, \end{array}$$

where $\varepsilon ={\left(j+1\right)}^{-1}$,

(23) $$J^{\pm }=ie^{\mp i\psi }\left[i\frac{\partial }{\partial \theta }\pm \cot\theta \frac{\partial }{\partial \psi }\mp \frac{1}{\sin\theta }\frac{\partial }{\partial \phi }\right]\;\;J^{0}=-i\frac{\partial }{\partial \psi }\;$$

and the notation A⊗B means,

(24) $$\left(A⊗B\right)\left(W_{f}^{j_{1}}W_{g}^{j_{2}}\right)=\left(AW_{f}^{j_{1}}\right)\left(BW_{g}^{j_{2}}\right).$$

Explicitly applying Equation (21) to the symbols of operators $\hat{f}$ and $\hat{g}$ and performing an integration and summation, one obtains the following symbolic expression for the symbol of their product

(25) $$W_{fg}^{j}\left(\Theta \right)\approx V\left(W_{f}^{j+I⊗J^{0}}\left(\Theta \right)W_{g}^{j-J^{0}⊗I}\left(\Theta \right)\right),$$

where the operator indices of each symbol are applied to the right or to the left according to (24). The above expression can be further simplified in the limit j≫1 and considering *j* as a continuous variable (see Section 4). However, the continuous limits for symbols $W_{f}^{j\;}\left(\Theta \right)$ are different for even and odd values of the index *j* due to the parity property (11) and (12). It is convenient to introduce the linear combinations

(26) $$\begin{array}{ccc} W_{f}^{j+\;}\left(\Theta \right) & = & W_{f}^{j\;}\left(\Theta \right)+W_{f}^{j+1\;}\left(\Theta \right), \end{array}$$

(27) $$\begin{array}{ccc} W_{f}^{j-\;}\left(\Theta \right) & = & {\left(-1\right)}^{j}\left(W_{f}^{j\;}\left(\Theta \right)-W_{f}^{j+1\;}\left(\Theta \right)\right), \end{array}$$

which are related through a phase shift,

(28) $$W_{f}^{j-\;}\left(\phi ,\theta ,\psi \right)=W_{f}^{j+\;}\left(\phi ,\theta ,\psi +\pi \right).$$

For instance, $W_{r_{k}}^{j+}\left(\Theta \right)=r_{k},$ for any (integer) value of the index *j*. The symbols (26) and (27) become smooth functions of *j* in the continuous limit, $W_{f}^{j\pm \;}\left(\Theta \right)\to W_{f}^{\pm \;}\left(\Theta ,j\right)$.

Of particular interests are symbols $W^{j\pm \;}\left(\Theta \right)$ with index *j* distributed in a vicinity, $1\ll \delta \ll j_{0},$ of some $j_{0}$. Actually, smooth and localized functions of $\left(\Theta ,j\right)$, with $\delta ∼j_{0}^{1/2}$ for $j_{0}\gg 1$ correspond to the so-called semi-classical states. Physically, such states are spread among several SO(3) invariant subspaces characterized by a large value of spin and localized in angle variables.

The Schrodinger equation

$$i\partial_{t}\hat{\rho }=\left[\hat{H},\hat{\rho }\right],$$

$\hat{H}$ being the Hamiltonian of the system, is mapped into the evolution equations for the Wigner functions

(29) $$i\partial_{t}W_{\rho }^{j}=W_{H\rho }^{j}-W_{\rho H}^{j}.$$

In the continuous limit and for initial semi-classical states, Equation (29) is reduced in the leading order on $j_{0}$ to the Liouville-type differential equations [12] (see also Appendix A) for $W_{\rho }^{\;\pm }\left(\Theta ,j\right)$,

(30) $$\partial_{t}W_{\rho }^{\;\pm }\left(\Theta ,j\right)\approx 2\left\{W_{H}^{\;\pm }\left(\Theta ,j\right),W_{\rho }^{\;\pm }\left(\Theta ,j\right)\right\},$$

where {.,.} are the Poisson brackets in the Darboux coordinates $\left(\left(j+1\right)\cos\theta ,\phi \right)$ and $\left(j,\psi \right)$, and $W_{H}^{\pm }$ are the corresponding symbols of the Hamiltonian. The above equation defines a classical evolution on the symplectic manifold isomorphic to the cotangent bundle $T^{*}S_{2}$, which corresponds to the co-adjoint orbit of the SE(3) group fixed by the values of the Casimir operators ${\hat{r}}^{2}=\hat{I}$ and $\hat{l}\cdot \hat{r}=0$. Thus, in the semi-classical limit, the Wigner functions $W_{\rho }^{\pm }\left(\Theta ,j\right)$ can be considered as distributions in a four-dimensional manifold $T^{*}S_{2}$ and Equation (30) determines “classical trajectories” $\left(\Theta^{cl}\left(t\right),j^{cl}\left(t\right)\right)$ for variable spin systems. A classical observable *f* can be associated either with $W_{f}^{+}\left(\Theta ,j\right)$ or $W_{f}^{-}\left(\Theta ,j\right)$; however, it is more convenient to choose $W_{f}^{+}\left(\Theta ,j\right)$ due to the relation (28). The explicit form of the Poisson brackets on $T^{*}S_{2}$ is given in Appendix A, Equation (A6). Strictly speaking, the real expansion parameter, used in transition from (29) to (30), is $j{\left(t\right)}^{-1}$ (for *j* initially localized close to $j_{0}\gg 1$). Therefore, the Liouville Equation (30) holds, while, on average over the distribution, $j\left(t\right)∼{\langle {\hat{l}}^{2}\rangle }^{1/2}\gg 1$.

The evolution of average value of an operator $\hat{f}$ evaluated according to the overlap relation (8) is convenient to rewrite in terms of symbols $W_{f}^{j\pm \;}\left(\Theta \right)$, as follows:

(31) $$\langle \hat{f}\left(t\right)\rangle =\sum_{j=0,1,2,\cdots }\frac{j+1}{16\pi^{2}}\int d\Theta W_{f}^{j+\;}\left(\Theta |t\right)\left(W_{\rho }^{j+}\left(\Theta \right)+{\left(-1\right)}^{j}W_{\rho }^{j-}\left(\Theta \right)\right),$$

where $W_{f}^{j+\;}\left(\Theta |t\right)$ is the symbol of the Heisenberg operator $\hat{f}\left(t\right)$. In the continuous limit, changing summation on *j* to integration, the contribution of the second term in the above equation becomes negligible. Then, in the spirit of TWA, considering (Θ,j) as dynamic variables, the average values of physical observables can be estimated according to (8)

(32) $$\langle \hat{f}\left(t\right)\rangle \approx \int_{0}^{\infty }dj\frac{j+1}{16\pi^{2}}\int d\Theta W_{f}^{+\;}\left(\Theta^{cl}\left(t\right),j^{cl}\left(t\right)\right)W_{\rho }^{+}\left(\Theta ,j\right),$$

where the integration is carried out on the initial conditions of the classical trajectories $j^{cl}\left(t\right)=j^{cl}\left(\Theta ,j|t\right)$, $\Theta^{cl}\left(t\right)=\Theta^{cl}\left(\Theta ,j|t\right)$.

Actually, since it is expected that $W_{\rho }^{+}\left(\Theta ,j\right)$ is sharply localized at $j∼j_{0}\gg 1$ (commonly the width of the *semiclassical* Wigner distribution is $∼\sqrt{j_{0}}$), Equation (32) can be well approximated as

(33) $$\langle \hat{f}\left(t\right)\rangle \approx \int_{-\infty }^{\infty }dj\frac{j+j_{0}+1}{16\pi^{2}}\int d\Theta W_{f}^{+}\left(\Theta ,j+j_{0}|t\right)W_{\rho }^{+}\left(\Theta ,j+j_{0}\right),$$

where

$$W_{f}^{+}\left(\Theta ,j+j_{0}|t\right)\equiv W_{f}^{+}\left(\Theta^{cl}\left(\Theta ,j+j_{0}|t\right),j^{cl}\left(\Theta ,j+j_{0}|t\right)\right),$$

and $W_{\rho }^{+}\left(\Theta ,j+j_{0}\right)$ is now centered at zero in the *j* axis. In addition, the semiclassical distributions $W_{\rho }^{j\pm }\left(\Theta \right)$ are approximately normalized

$$\sum_{j=0,1,2,\ldots }^{\infty }\frac{j+1}{16\pi^{2}}\int d\Theta W_{\rho }^{j\pm }\left(\Theta \right)\approx 1.$$

As well as in cases of lower dimensional manifolds, it cannot be expected that propagation along distinguishable classical trajectories (there is no trajectory crossing), originated at every phase-space point of the initial distribution, are able to describe a long-time non-harmonic dynamics [69,70,71]. However, as it will be shown below, there is a natural form to improve the time-validity of TWA for initial semi-classical states of variable spin systems.

It is worth emphasizing that only $W_{\rho }^{\;\pm }\left(\Theta ,j\right)$ combinations satisfy the Liouville evolution Equation (30). Thus, the symbols $W_{f}^{+\;}\left(\Theta ,j|t\right)$ should be associated with the evolving classical observables according to (31) and (32).

## 4. Asymptotic Quantization and Discretization Procedure

We start noting that the integral (32) may describe only a destructive dynamic interference corresponding to the initial stage of non-harmonic quantum evolution (collapse time). Such a behavior is due to a *continuous* superposition of independent classically propagated infinitesimally close fractions of the initial distribution. Several discretization procedures [51,52] have been proposed in order to overcome this problem and to extend the time validity of TWA. Among them, one can mention a semi-classical discretization based on propagating a single trajectory out of each Plank cell in a flat phase-space [44], application of the discrete Wigner function method [72] to a collection of 1/2-spin systems [45,46,47,48,49,50].

The form of phase-space evaluation of average values in variable spin system (31) suggests an intuitive way for a partial discretization of the initial distribution in the semi-classical limit. In our approximation, the discrete index *j*, which originally appeared in the expansion (9) for labeling the angular momentum sectors, is now considered as a classical dynamic variable. Then, having applied the star-product (25) to the symbols (26) and (27), we immediately arrive in the continuous limit at the following asymptotic form of the star-product

(34) $$\begin{array}{ccc} W_{fg}^{\pm }\left(\Theta ,j\right) & \approx & e^{-\varepsilon J^{0}⊗J^{0}-\frac{\varepsilon }{2}\left(J^{+}⊗J^{-}-J^{-}⊗J^{+}\right)}e^{\partial_{j}⊗J^{0}-J^{0}⊗\partial_{j}}\left[W_{f}^{\pm }\left(\Theta ,j\right)W_{g}^{\pm }\left(\Theta ,j\right)\right] \end{array}$$

(35) $$\begin{array}{ccc} & \approx & W_{f}^{\pm }\left(\Theta ,j\right)\;*W_{g}^{\pm }\left(\Theta ,j\right), \end{array}$$

where $e^{\partial_{j}⊗J^{0}}f\left(j\right)=f\left(j+I⊗J^{0}\right)$. It is worth noting that the star-product in the form (34) is applicable only to the classical observables, i.e., (26) and (27) symbols.

Strictly speaking, Equation (34) should be applied to functions with the index *j* distributed in a broad vicinity, δ≫1, of some $j_{0}\gg 1$, so that $\varepsilon \approx {\left(j_{0}+1\right)}^{-1}$ and $\partial_{j}∼\delta^{-1}$. However, a direct application of Equation (34) to the generators of the se(3) algebra ($\hat{l}$, $\hat{r}$) shows a good correspondence between the exact commutation relations and their counterparts reconstructed through the asymptotic star-product:

$$\begin{array}{ccc} W_{\left[l_{k},l_{n}\right]}^{+}\left(\Theta ,j\right) & = & i\epsilon_{knm}W_{l_{m}}^{+}\left(\Theta ,j\right)+O\left(\varepsilon \right)\\ W_{\left[l_{k},r_{n}\right]}^{+}\left(\Theta ,j\right) & = & i\epsilon_{knm}W_{r_{m}}^{+}\left(\Theta ,j\right)+O\left(\varepsilon \right),\;W_{\left[r_{k},r_{n}\right]}^{+}\left(\Theta ,j\right)=0, \end{array}$$

where

(36) $$W_{\left[f,g\right]}\left(\Theta ,j\right)=W_{f}\left(\Theta ,j\right)\;*\;W_{g}\left(\Theta ,j\right)-W_{g}\left(\Theta ,j\right)\;*\;W_{f}\left(\Theta ,j\right).$$

It is worth noting that asymptotically the operator corresponding to the classical observable $(j+j_{0})/2$ is conjugated to the operator corresponding to the phase $e^{i\psi }$,

(37) $$W_{\left[\left(j+j_{0}\right)/2,e^{i\psi }\right]}^{+}\left(\Theta ,j\right)\approx W_{e^{i\psi }}^{+}\left(\Theta ,j\right).$$

In a sense, $(j+j_{0})/2$ and $e^{i\psi }$ can be seen as action-angle variables. Here, we consider $\left(j+1\right)/2$ as a physical variable representing the classical angular momentum, as $W_{l_{k}}^{+}\left(j,\Theta \right)\approx \frac{j+1}{2}\;n_{k}$, according to (16) and (17).

Making use of the asymptotic form of the star-product (34), we can discretize back the variable *j* following the ideas of deformation quantization. According to the general procedure [66], we solve the eigenvalue equation

$$i\partial_{\tau }U=\left(j+j_{0}\right)\;*\;U\approx \;\left(j+j_{0}-i\partial_{\psi }\right)U,$$

$$U\left(\left.j+j_{0},\Theta \right|\tau =0\right)=1,$$

where the expression (34) was employed. A direct expansion of the solution

$$U\left(\left.j+j_{0},\Theta \right|\tau \right)=e^{-i\;\left(j+j_{0}\right)\tau },\;$$

in the Fourier series yields

$$e^{-i\left(j+j_{0}\right)\tau }=\sum_{L=-\infty }^{\infty }\Pi_{L}\left(j\right)e^{m\pi i\left(j+j_{0}\right)}e^{-i\left(L+j_{0}\right)\tau },\;\left(2m-1\right)\pi <\tau <\left(2m+1\right)\pi ,$$

where

(38) $$\Pi_{L}\left(j\right)=\frac{\sin\pi \left(j-L\right)}{\pi \left(j-L\right)}.$$

Since the Wigner distributions $W_{\rho }^{\pm }\left(\Theta ,j+j_{0}\right)$ have compact supports, with width $∼\sqrt{j_{0}}\gg 1$, we can make use of the Whittaker–Shannon–Kotelnikov sampling theorem [73,74,75], approximating

(39) $$\begin{array}{ccc} & & \frac{j+j_{0}+1}{16\pi^{2}}W_{f}^{+}\left(\Theta ,j+j_{0}|t\right)W_{\rho }^{\pm }\left(\Theta ,j+j_{0}\right)\\ & \approx & \sum_{L=-\infty }^{\infty }\frac{L+j_{0}+1}{16\pi^{2}}W_{f}^{+}\left(\Theta ,L+j_{0}|t\right)W_{\rho }^{\pm }\left(\Theta ,L+j_{0}\right)\;\Pi_{L}\left(j\right). \end{array}$$

It is worth noting that, in case of Gaussian function of width $∼\sqrt{j_{0}}$, the error of discrete sampling with (38) is of order ∼erfc$\left(\pi \sqrt{j_{0}}\right)$ [76].

However, a direct discretization (39) of the semi-classical expression (33) is not sufficient for an efficient simulation of the quantum dynamics through phase-space trajectories since the overlap between the classically evolved observable $W_{f}^{j+\;}\left(\Theta |t\right)$ and the branch $W_{\rho }^{j-}\left(\Theta \right)$ of distribution would be missed. It is worth recalling that $W_{\rho }^{+}\left(\Theta ,j\right)$ and $W_{\rho }^{-}\left(\Theta ,j\right)$ have maxima at different points of the phase space (shifted in π on ψ). In order to correct this problem, we rewrite Equation (31)

(40) $$\begin{array}{ccc} \langle \hat{f}\left(t\right)\rangle & = & \Sigma_{+}\left(t\right)+P\Sigma_{-}\left(t\right) \end{array}$$

(41) $$\begin{array}{ccc} \Sigma_{\pm }\left(t\right) & = & \sum_{j=0,1,2,\cdots }\frac{j+1}{16\pi^{2}}\int d\Theta W_{f}^{j+\;}\left(\Theta |t\right)W_{\rho }^{j\pm }\left(\Theta \right), \end{array}$$

where P is the parity operator defined according to

$$P\sum_{j=0,1,2,..}a_{j}=\sum_{j=0,1,2,..}{\left(-1\right)}^{j}a_{j}.$$

Then, considering the semi-classical evolution of $W_{f}^{+\;}\left(\Theta ,j\right)$ in the continuous limit and applying a subsequent discretization procedure (39)–(41), we arrive at the following discretized version of TWA for variable spin systems,

(42) $$\langle \hat{f}\left(t\right)\rangle \approx \sum_{L=-\infty }^{\infty }\frac{L+j_{0}+1}{16\pi^{2}}\int d\Theta W_{f}^{+}\left(\Theta ,L+j_{0}|t\right)\left(W_{\rho }^{+}\left(\Theta ,L+j_{0}\right)+{\left(-1\right)}^{L}W_{\rho }^{-}\left(\Theta ,L+j_{0}\right)\right).$$

The above equation is just a convolution of the evolving classical observable with a linear combination of the initial distribution $W_{\rho }^{+}$ at (ϕ,θ,ψ) and (ϕ,θ,ψ+π), evaluated at the equiseparated points along the “action” variable *j*. A naive direct discretization of the continuous approximation (32) leads to an incomplete description of the quantum dynamics in the semiclassical limit.

## 5. Examples

### 5.1. Rigid Rotor in an External Field

Let us consider the following Hamiltonian governing the evolution of quantum rigid rotor in an external field along the *z*-axis,

(43) $$\hat{H}={\hat{l}}^{2}-g{\hat{z}}^{2},$$

where $\hat{z}$ is the *z*-component of the orientation operator (13), and ${\hat{l}}^{2}$ is the square angular momentum operator. The Hamiltonian (43) possesses the SO(3) symmetry but cannot be reduced to a finite number of spin systems.

The symbol of the Hamiltonian in the continuous limit at the principal order on *j* has the form

(44) $$W_{H}^{+}\left(\Theta ,j\right)\approx \frac{j}{2}\left(\frac{j}{2}+1\right)-g\sin^{2}\theta \cos^{2}\psi$$

and leads to the following equations of motion:

(45) $$\begin{array}{ccc} \partial_{t}\phi & = & \frac{4g}{j+1}\cos\theta \cos^{2}\psi \\ \partial_{t}\theta & = & -\frac{g}{j+1}\sin2\theta \sin2\psi \\ \partial_{t}\psi & = & j+1-\frac{4g}{j+1}\cos^{2}\theta \cos^{2}\psi \\ \partial_{t}j & = & -2g\sin^{2}\theta \sin2\psi . \end{array}$$

**Figure 1:**
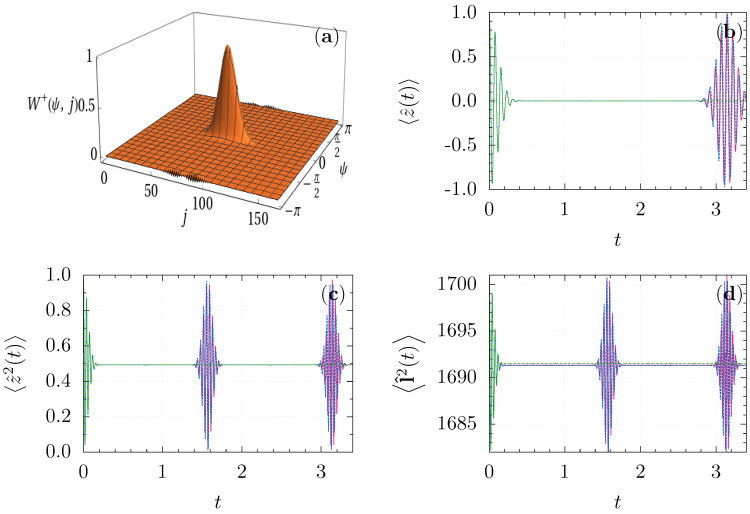
(**a**) The marginal distribution $W_{\rho }^{+}\left(\psi ,j\right)$ corresponding to the state (46); (**b**–**d**) evolution of $\langle \hat{z}\left(t\right)\rangle$, $\langle {\hat{z}}^{2}\left(t\right)\rangle$, $\langle {\hat{l}}^{2}\left(t\right)\rangle$ generated by the Hamiltonian (43) with g=20, for the initial state (46) with $r^{2}=81$, $\varphi_{0}=\psi_{0}=0$, $\vartheta_{0}=\pi /2$: exact evolution (solid magenta line), continuous $T^{*}S_{2}$ TWA (32) (dashed green line), discrete TWA (42) (dashed blue line).

As an initial state, we consider a weighted superposition of *l*-spin coherent states $|l;\vartheta_{0},\varphi_{0}\rangle$

(46) $$|\Psi (0)\rangle =\frac{1}{\sqrt{\cosh r^{2}}}\sum_{l=0}^{\infty }e^{-il\psi_{0}}\frac{r^{2l}}{\sqrt{(2l)!}}|l;\vartheta_{0},\varphi_{0}\rangle ,$$

where $r^{2}\gg 1$. The Wigner distribution $W_{\rho }^{+}\left(\Theta ,j\right)$ corresponding to the state (46) can be approximated as

$$\begin{array}{ccc} W_{\rho }^{+}\left(\Theta ,j\right) & \approx & \frac{r^{2j}}{\Gamma \left(j+2\right)\cosh r^{2}}\frac{1}{\sin\omega /2}\partial_{\omega }\left(\frac{\sin^{2}\left(\left(j+1\right)\omega /2\right)}{\sin\omega /2}\right),\\ \cos\frac{\omega }{2} & = & \frac{1}{\sqrt{2}}\left(\cos\frac{\theta }{2}\cos\frac{\phi +\psi }{2}+\sin\frac{\theta }{2}\cos\frac{\phi -\psi }{2}\right), \end{array}$$

and is localized in $\vartheta ∼\vartheta_{0}=\pi /2$, $\varphi ∼\varphi_{0}=0$ by construction, with absolute fluctuations $\delta \vartheta ∼\delta \varphi ∼r^{-1}$. In addition, $W_{\rho }^{+}\left(\Theta ,j\right)$ is localized at $j∼r^{2}$ with the fluctuation δj∼r. Thus, the phase ψ is also localized with $\delta \psi ∼r^{-1}$, as it is an observable conjugated to *j*, according to (37), see Figure 1a where the marginal distribution

(47) $$W_{\rho }^{+}\left(\psi ,j\right)=\frac{j+1}{8\pi }\int_{0}^{2\pi }d\phi \int_{0}^{\pi }\sin\theta d\theta W_{\rho }^{+}\left(\Theta ,j\right)$$

is plotted. The same reasoning is applicable to $W_{\rho }^{-}\left(\Theta ,j\right)$ due to the relation (28). This means that the state (46) is a semi-classical state on the manifold $T^{*}S_{2}$.

We have numerically tested the approximation (42) by computing the averages of $\hat{z}\left(t\right)$, ${\hat{z}}^{2}\left(t\right)$, and ${\hat{l}}^{2}\left(t\right)$. For numerical simulations, we have used an adaptive sampling technique that allows us to sample the angular variables in the regions where the initial Wigner function is located, for every fixed value of *j*. The sampling method is based on an algorithm [77] for numerical integration of multivariate functions and implemented by a modification of the routine *cubature* [78]. For $r^{2}=81$, g=20, we take on average 7892 points inside $S_{3}$ sphere for each value of integer $j\in \left[40,160\right]$. The errors obtained in the estimation of the expectation values are lower than 0.0045% at t=0. The differential equations were solved by a variable step-size Runge–Kutta method of order 9(8) [79]. The size of the step was adapted to keep the relative errors estimated by the method lower than $10^{-6}$.

In Figure 1b–d, we plot the corresponding averages in comparison with the exact calculations and the continuous TWA approximation (33). One can appreciate that Equation (33) coincides with the exact calculations only within the initial collapse, i.e., for times gt≪1. Conversely, Equation (42) describes very well the evolution of the observables inclusively for much longer intervals that include several revivals, gt∼π. For even longer times, $gt∼r^{2}$, our approximation starts to deviate from the exact solution, failing to capture the oscillation dephasing and deformations of envelopes of the revivals (although the condition j(t)≫1 still holds).

It is worth noting that, in contrast to planar pendulum models, described by periodic Hamiltonians of the form

$$\hat{H}={\hat{p}}^{2}+V\left(\hat{x}\right),\;V\left(x+a\right)=V\left(x\right),\;a=const,$$

where $\hat{p}$ and $\hat{x}$ are the standard momentum and position operators, a phase-space description of the rigid rotor in an external field is not trivial. For instance, the standard phase-space analysis of the Hamiltonian (43) in $C^{2}⊗C^{2}$, and application of the corresponding TWA [19], faces considerable technical difficulties. In particular, the Schwinger (two-mode) representation of $\hat{z}$ operator,

$$\hat{z}=\frac{1}{\sqrt{\hat{a}{\hat{a}}^{†}+{\hat{b}}^{†}\hat{b}}}\left({\hat{a}}^{†2}+{\hat{b}}^{†}\right)\frac{1}{\sqrt{\hat{a}{\hat{a}}^{†}+{\hat{b}}^{†}\hat{b}}},$$

is quite inconvenient for the H(1)×H(1) phase-space mapping [53,54,55,56], and hides the intrinsic SO(3) symmetry of the direction operator (13) and (14).

### 5.2. Spin–Spin Interaction

As another non-trivial example, we consider the following Hamiltonian:

(48) $$\hat{H}={\hat{l}}_{1}\cdot {\hat{l}}_{2}+\lambda {\hat{l}}_{z1},$$

describing an integer spin–spin interaction in the presence of an external non-uniform magnetic field.

Within the framework of our approach, the symbol of the Hamiltonian in the continuous limit has the form

$$W_{H}^{+}\left(\Theta ,j\right)=C\left(j\right)+\lambda A\left(j\right)\sin\theta \cos\psi +\lambda B\left(j\right)\cos\theta ,$$

where A(j), B(j) and C(j) are functions of *j* given in Appendix B.

Taking into account the classical equations of motion on $T^{*}S_{2}$ (see Appendix B), we compute the evolution of the first spin magnetization $\langle {\hat{l}}_{x1}\left(t\right)\rangle$ according to the general procedure (42), where the symbol of ${\hat{l}}_{x1}$ is

$$W_{l_{x1}}^{+}\left(\Theta ,j\right)=A\left(j\right)\left(\sin\phi \sin\psi -\cos\phi \cos\theta \cos\psi \right)+B\left(j\right)\cos\phi \sin\theta .$$

As the initial state, we consider the product of spin coherent states in *x*- and *y*-directions,

(49) $$|\Psi \left(0\right)\rangle =|L_{1};\varphi_{1}=0,\vartheta_{1}=\pi /2\rangle ⊗|L_{2};\varphi_{2}=\pi /2,\vartheta_{2}=\pi /2\rangle .$$

**Figure 2:**
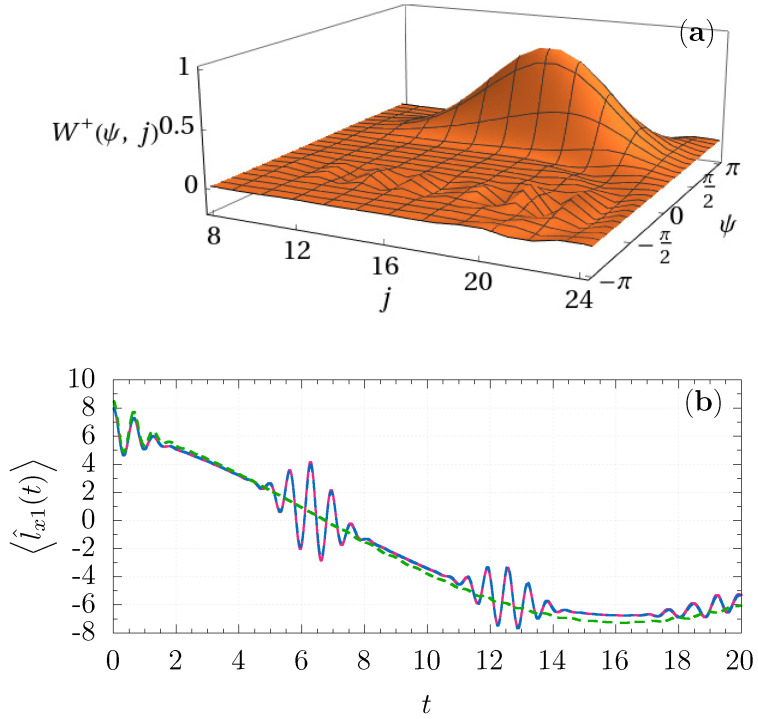
(**a**) The marginal distribution $W_{\rho }^{+}\left(\psi ,j\right)$ corresponding to the state (49); (**b**) evolution of $\langle {\hat{l}}_{x1}\left(t\right)\rangle$ generated by the Hamiltonian (48) with λ = 0.2, the initial state is $|L_{1}=8;\varphi_{1}=0,\vartheta_{1}=\pi /2\rangle |L_{2}=4;\varphi_{2}=\pi /2,\vartheta_{2}=\pi /2\rangle$: exact evolution (solid magenta line), SO(3)×SO(3) TWA (51) (dashed green line), discrete TWA (42) (dashed blue line).

The general expression for the Wigner function of the state (49) is quite cumbersome, but its localization property at $j∼2L_{1}$ for $L_{1}\gg L_{2}$ follows from the marginal distribution

$$\int_{0}^{2\pi }\frac{d\psi }{2\pi }W_{\rho }^{+}\left(\Theta ,j\right)∼e^{-{\left(j/2-L_{1}\right)}^{2}/L_{2}}W_{\rho }\left(\phi ,\theta ;j\right),$$

where $W_{\rho }\left(\phi ,\theta ;j\right)=\langle L=j/2;\varphi =0,\vartheta =\pi /2|{\hat{w}}_{L=j/2}\left(\phi ,\theta \right)|L=j/2;\varphi =0,\vartheta =\pi /2\rangle$ is the standard SO(3) Wigner function (19). The localization on the angle ψ follows from its complementarity to the variable *j*, in the same way as in the rotor case. The marginal distribution (47) corresponding to the state (49) is plotted in Figure 2a.

It is worth noting that this system has SO(3)×SO(3) symmetry (for integer spins), so that the operators acting in the Hilbert space of two-spin system can be mapped into distributions in $S_{2}\times S_{2}$ using the following the mapping kernel,

(50) $$\hat{w}\left(\Omega_{1},\Omega_{2}\right)={\hat{w}}_{1}\left(\Omega_{1}\right)⊗{\hat{w}}_{2}\left(\Omega_{2}\right),$$

where $\hat{w}\left(\Omega \right)$, $\Omega =\left(\phi ,\theta \right)$ is defined in (19). In the limit of large spins, the Hamiltonian dynamics can be treated semi-classically [20,21,22,23,27]. The equation of motion for the Wigner function of the whole system ${\tilde{W}}_{\rho }\left(\Omega_{1},\Omega_{2}\right)$ takes the form:

$$\begin{array}{ccc} \partial_{t}{\tilde{W}}_{\rho } & \approx & \frac{1}{L_{1}+1/2}{\left\{{\tilde{W}}_{H},{\tilde{W}}_{\rho }\right\}}_{1}+\frac{1}{L_{2}+1/2}{\left\{{\tilde{W}}_{H},{\tilde{W}}_{\rho }\right\}}_{2},\\ {\tilde{W}}_{H}\left(\Omega_{1},\Omega_{2}\right) & = & \sqrt{L_{1}\left(L_{1}+1\right)L_{2}\left(L_{2}+1\right)}n_{1}\cdot n_{2}+\lambda \sqrt{L_{1}\left(L_{1}+1\right)}n_{z1}, \end{array}$$

where the canonical variables defining the Poisson brackets ${\left\{..,..\right\}}_{1,2}$ are $\left(\cos\theta_{1,2},\phi_{1,2}\right)$, and the average values are computed according to the standard TWA,

(51) $$\langle \hat{f}\left(t\right)\rangle \approx \frac{\left(2L_{1}+1\right)\left(2L_{2}+1\right)}{16\pi^{2}}\int d\Omega_{1}d\Omega_{2}{\tilde{W}}_{f}(\left(\Omega_{1}^{cl}\left(t\right),\Omega_{2}^{cl}\left(t\right)\right){\tilde{W}}_{\rho }\left(\Omega_{1},\Omega_{2}\right),$$

$\Omega_{1}^{cl}\left(t\right)$ and $\Omega_{2}^{cl}\left(t\right)$ being classical trajectories on $S_{2}\times S_{2}$.

For numerical simulations, we used the same adaptive method as in the case of the rotor. For the spin–spin system, $L_{1}=8$, $L_{2}=4$, λ=0.2, $j\in \left[8,24\right],$ the average number of samples is 26,885. The errors in the estimation of the expectation values are lower than 0.0008% at t=0.

Comparing the results obtained from (42) and (51), one can observe that the proposed discretization leads to a good coincidence with the exact results significantly beyond the validity of the standard TWA in the framework of Stratonovich–Weyl correspondence. Actually, our approximation describes well the effect of partial revivals produced by the nonlinear term ${\hat{l}}_{1}\cdot {\hat{l}}_{2}$ at the scale t∼2π, but falling at $t∼2L_{1}$ (independently on the external field coupling constant λ). The standard TWA breaks down already after the first collapse at t≲1.

## 6. Conclusions

The semi-classical map (1)–(3) of density matrices of a variable spin system into distributions on four-dimensional symplectic manifold allows for approximating the evolution of such quantum systems in terms of effective classical dynamics on $T^{*}S_{2}$. The advantage of the map (3) with respect to the standard SU(2)/SO(3) case [57] consists of the possibility of a faithful representation of operators whose action is not restricted to a single SU(2) invariant subspace. In addition, only four Hamilton equations are sufficient to determine the evolution of classical observables for any value of the total angular momentum. However, the simplest Truncated Wigner Approximation suffers from the same intrinsic defects as in the Heisenberg–Weyl and SU(2) symmetries: it describes well only the short-time evolution of the typical observables of the system (which may include, e.g., generators of SE(3) group). In order to extend the validity of TWA, while still keeping the idea of classical propagation, we propose to “quantize” back one of the classical dynamic variables, *j*, which can be considered to some extent as an “action” (actually representing possible values of the classical spin size). We perform such a “quantization” by using the asymptotic form of the star-product within the framework of deformation quantization, leading to a natural discretization of the variable *j*, and, thus, all of the distributions appearing in the theory, corresponding both to states and observables. A certain subtlety of the proposed method consists of taking into account the parity problem originated from the decomposition of the mapping kernel (4) in the basis of the tensor operators (5). In addition, it results that the obtained discretization of initial distributions with compact support, describing the so-called semi-classical states, is in direct accordance with the famous sampling theorem. This allows the form of calculation of average values to be immediately discretized, basically starting the classical trajectories only at certain points of the initial distribution. The result of such an approach is surprisingly good, as shown in Figure 1 and Figure 2. It is worth noting that the discrete sampling procedure in general is not obvious at all. For instance, applying more sophisticated discretization methods, like, e.g., the adaptive discretization, one obtains much worse results than by following the simple recipe (42). Actually, Equation (42) describes very well all interference effects, such as, e.g., revivals of quantum oscillations that appear due to superpositions of subspaces with different values of the index *j*, appearing in the exact calculations (8).

**Figure 3:**
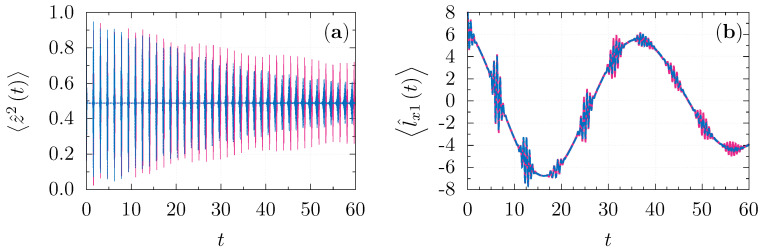
(**a**) Long-time evolution of $\langle {\hat{z}}^{2}\left(t\right)\rangle$ generated by the Hamiltonian (43) with g=20, for the initial state (46) with $r^{2}=49$, ϕ=0,ψ=0,θ=π/2; (**b**) evolution of $\langle {\hat{l}}_{x1}\left(t\right)\rangle$ generated by the Hamiltonian (48) with λ = 0.2, the initial state is $|L_{1}=8;\varphi_{1}=0,\vartheta_{1}=\pi /2\rangle |L_{2}=4;\varphi_{2}=\pi /2,\vartheta_{2}=\pi /2\rangle$: exact evolution (solid magenta line), discrete TWA (42) (dashed blue line).

The range of applicability of the discretized TWA is considerably longer than the standard semiclassical time $\tau_{sem}$, $gt≲j_{0}^{\beta }\tau_{sem}$, β>0, where $j_{0}\gg 1$ is the average initial total angular moment. For second degree Hamiltonians, similar to (43) and (48), the leading corrections to the Liouville Equation (30) are of the order $j_{0}^{-1}$, while the principal term (the Poisson bracket on $T^{*}S_{2}$) is $∼j_{0}$. Actually, the Moyal Equation (29) has in this case the following structure

(52) $$\partial_{t}W_{\rho }^{\;}=\left(j_{0}L_{0}+\;j_{0}^{-1}L_{2}\right)W_{\rho }^{\;}+O\left(j_{0}^{-2}\right),$$

where $L_{0}$ is a first order differential operator and $L_{2}$ contains higher-degree derivatives. Dropping the correction terms $j_{0}^{-1}L_{2}$ in the continuous TWA leads to neglecting all of the commutators of order $j_{0}^{0}=1$ that appear in the exponent of the formal propagator corresponding to Equation (52). As a result, the description of quantum dynamics occurring in the time-scale gt≳1 is inaccessible in the semiclassical treatment (32). In practice, the standard TWA [19] breaks down even for shorter time intervals and is unable to describe any genuine quantum effect such as, for example, quantum revivals. It seems that the discretization (42) allows physical processes caused by the interferences between different *j*-sectors (9) to be emulated until $gt≲j_{0}$. This is really not surprising, since similar effects take place in almost all nonlinear quantum systems with discrete spectra as a result of a specific composition of some *discrete* constituents [80]. The difficulty consists, as we have mentioned above, in finding an appropriate discretization of the classical phase-space. In Figure 3a,b, the long time evolution of the rotor (43) and spin observables (48) are shown. One can clearly observe the region of time validity of our approximation.

The present approach is also extendable to half-integer spins, although the calculations become more involved. In such a case, four linear combinations of the Weyl symbols $W_{f}^{j\;}\left(\Theta \right),$ similar to (26) and (27), should be introduced in order to follow the same procedure as in Section 3 and Section 4. This problem will be considered elsewhere in application to dissipative and pumped down conversion processes.

Recently, a generalized TWA was proposed in [81] for the description of the dynamics of many coupled spins. In this approach, every spin is considered as a discrete variable, i.e., there is no relation to any classical phase-space. Thus, a set of $2\left({\left(2L+1\right)}^{2}-1\right)$ coupled (first-order) differential equations should be solved even for two interacting spins *L*. In addition, the rigid rotor evolution in external fields cannot be treated applying the technique [81].

Unfortunately, the map (1)–(4) cannot be used as a faithful (one-to-one map) classical representation of the *N*-spin, N≥3 system. However, some global properties of multi-spin systems can be analyzed with our method by using the Schur–Weyl duality [82] and averaging over invariant subspaces of the same dimensions.

Finally, we note that the developed approach can, in principle, be applied to any of the *s*-parametrized maps [12]. However, the Moyal equation for non self-dual distributions $W_{\rho }^{(s)\;}$ contains terms of order one (on the semiclassical parameter), i.e., it has the form

$$\partial_{t}W_{\rho }^{(s)\;}=\left(j_{0}L_{0}+sL_{1}+\;j_{0}^{-1}L_{2}\right)W_{\rho }^{(s)\;}+O\left(j_{0}^{-2}\right),$$

where $L_{1}$ is a differential operator already containing higher than first degree derivatives. Thus, one may expect that the TWA for $W_{\rho }^{(s\neq 0)\;}$, which considers the action only of $L_{0}$, is less precise than for $W_{\rho }^{(s=0)\;}$. 

## Data Availability

Not applicable.

## Appendix A

Since the continuous limits are different for symbols $W_{f}^{j\;}\left(\Theta \right)$ with even and odd values of the index *j*, Equation (25) should be expanded separately for $W_{fg}^{j\;(e)}\left(\Theta \right)$ and $W_{f}^{j(o)\;}\left(\Theta \right).$ For even values of the index *j*, Equation (25) takes the form

(A1) $$W_{fg}^{j\;(e)}\left(\Theta \right)\approx V\left(W_{f}^{j+I⊗J^{0}\;\left(e\right)}\left(\Theta \right)W_{g}^{j-J^{0}⊗I\;\left(e\right)}\left(\Theta \right)+W_{f}^{j+I⊗J^{0}\;\left(o\right)}\left(\Theta \right)W_{g}^{j-J^{0}⊗I\;\left(o\right)}\left(\Theta \right)\right),$$

while, for odd *j*, it becomes

(A2) $$W_{fg}^{j+1\;(o)}\left(\Theta \right)\approx V\left(W_{f}^{j+1+I⊗J^{0}\;\left(e\right)}\left(\Theta \right)W_{g}^{j+1-J^{0}⊗I\;\left(o\right)}\left(\Theta \right)+W_{f}^{j+1+I⊗J^{0}\;\left(o\right)}\left(\Theta \right)W_{g}^{j+1-J^{0}⊗I\;\left(e\right)}\left(\Theta \right)\right),$$

where V is defined in (22).

In the semi-classical limit, when j≫1 is considered continuous, the direct expansion of the above equations gives

(A3) $$\begin{array}{ccc} W_{f}^{j+I⊗J^{0}\;\left(e\right)}\left(\Theta \right) & \approx & \left(I+\partial_{j}⊗J^{0}\right)W_{f}^{j\;(e)}\left(\Theta \right),\\ W_{f}^{j+1+I⊗J^{0}\;\left(e\right)}\left(\Theta \right) & \approx & \left(I+\partial_{j}⊗\left(I+J^{0}\right)\right)W_{f}^{j\;(e)}\left(\Theta \right),\\ W_{f}^{j+I⊗J^{0}\;\left(o\right)}\left(\Theta \right) & \approx & \left(I+\partial_{j}⊗\left(I-J^{0}\right)\right)W_{f}^{j+1\;(o)}\left(\Theta \right),\\ W_{f}^{j+1+I⊗J^{0}\;\left(o\right)}\left(\Theta \right) & \approx & \left(I+\partial_{j}⊗J^{0}\right)W_{f}^{j+1\;(o)}. \end{array}$$

Substituting (A1)–(A3) into the equations of motion

(A4) $$\begin{array}{ccc} i\partial_{t}W_{\rho }^{j\;(e)} & = & W_{H\rho }^{j\;(e)}\left(\Theta \right)-W_{\rho H}^{j\;(e)}\left(\Theta \right), \end{array}$$

(A5) $$\begin{array}{ccc} i\partial_{t}W_{\rho }^{j+1\;(o)} & = & W_{H\rho }^{j+1\;(o)}\left(\Theta \right)-W_{\rho H}^{j+1\;(o)}\left(\Theta \right), \end{array}$$

one obtains

$$\begin{array}{ccc} i\partial_{t}W_{\rho }^{j\;(e)} & \approx & 2\left\{W_{H}^{j\;(e)},W_{\rho }^{j\;(e)}\right\}+2\left\{W_{H}^{j+1\;(o)},W_{\rho }^{j+1\;(o)}\right\},\\ i\partial_{t}W_{\rho }^{j+1\;(o)} & \approx & 2\left\{W_{H}^{j\;(e)},W_{\rho }^{j+1\;(o)}\right\}+2\left\{W_{H}^{j+1\;(o)},W_{\rho }^{j\;(e)}\right\}, \end{array}$$

where

(A6) $$\begin{array}{ccc} \left\{.,.\right\} & = & -\frac{\cot\theta }{j+1}\left(\partial_{\theta }⊗\partial_{\psi }-\partial_{\psi }⊗\partial_{\theta }\right)\\ & & +\frac{1}{(j+1)\sin\theta }\left(\partial_{\theta }⊗\partial_{\phi }-\partial_{\phi }⊗\partial_{\theta }\right)+\left(\partial_{\psi }⊗\partial_{j}-\partial_{j}⊗\partial_{\psi }\right), \end{array}$$

are the Poisson brackets on $T^{*}S_{2}$. Introducing $W^{\;\pm }\left(\Theta ,j\right)$ symbols according to (26) and (27), we immediately arrive at the Liouville form (30).

## Appendix B

The functions A(j), B(j) and C(j) have the form

$$\begin{array}{ccc} A(j) & = & \frac{1}{j+1}\sqrt{\left({\left(L_{1}+L_{2}+1\right)}^{2}-{\left(\frac{j+1}{2}\right)}^{2}\right)\left({\left(\frac{j+1}{2}\right)}^{2}-{\left(L_{1}-L_{2}\right)}^{2}\right)},\\ B(j) & = & \frac{1}{\sqrt{j\left(j+2\right)}}\left(\frac{j}{2}\left(\frac{j}{2}+1\right)-L_{2}\left(L_{2}+1\right)+L_{1}\left(L_{1}+1\right)\right),\\ C(j) & = & \frac{1}{2}\left(\frac{j}{2}\left(\frac{j}{2}+1\right)-L_{1}\left(L_{1}+1\right)-L_{2}\left(L_{2}+1\right)\right). \end{array}$$

The classical equations of motion corresponding to the Hamiltonian (48) are

$$\begin{array}{ccc} \partial_{t}\phi & = & \frac{2\lambda B(j)}{j+1}-\frac{2\lambda A(j)}{j+1}\cot\theta \cos\psi ,\\ \partial_{t}\theta & = & \frac{2\lambda A(j)}{j+1}\cos\theta \sin\psi ,\\ \partial_{t}\psi & = & \frac{2\lambda A(j)}{j+1}\frac{\cos^{2}\theta }{\sin\theta }\cos\psi +\frac{j+1}{2}+\lambda \left(\frac{j+1}{\sqrt{j(j+2)}}-2B\left(j\right)\frac{4j+2j^{2}+1}{\left(j+2\right)\left(j+1\right)}\right)\cos\theta ,\\ & & +\frac{2\lambda A\left(j\right)\left[{\left(j+1\right)}^{4}-16{\left(\left(L_{1}+L_{2}+1\right)\left(L_{1}-L_{2}\right)\right)}^{2}\right]\sin\theta \cos\psi }{\left({\left(j+1\right)}^{2}-4{\left(L_{1}-L_{2}\right)}^{2}\right)\left({\left(j+1\right)}^{2}-4{\left(L_{1}+L_{2}+1\right)}^{2}\right)\left(j+1\right)},\\ \partial_{t}j & = & 2\lambda A(j)\sin\theta \sin\psi . \end{array}$$
